# Supplementary material for: Stereospecific lasofoxifene derivatives reveal the interplay between estrogen receptor alpha stability and antagonistic activity in ESR1 mutant breast cancer cells
Source: eLife. 2022 May 16;11:e72512. doi: 10.7554/eLife.72512 (PMC9177151; doi:10.7554/eLife.72512)
Supplement: Figure 2—source data 1. — Data were normalized by cell count. Antiestrogens are categorized as stabilizers if their maximum signal for WT ERα at 5 µM is greater than 1.5, neutral if between 0.9 and 1.1, and degrader if less than 0.9 in this assay. [file elife-72512-fig2-data1.docx]

**Figure 2-source data 1:** Ligand and Mutational Influences on Estrogen Receptor Alpha Cellular Turnover after 24 Hours. Data were normalized by cell count. Antiestrogens are categorized as stabilizers if their maximum signal for WT ERα at 5 µM is greater than 1.5, neutral if between 0.9 and 1.1, and degrader if less than 0.9 in this assay.

| **Hormone** | | | | |
| --- | --- | --- | --- | --- |
| **Ligand** | **IC_50_ (nM)** | **R^2^** | **Maxima (5 µM)** | |
| **Estradiol (E2)** | | | | |
| *WT* | 0.12 ± 0.02 | 0.806 | 0.68 ± 0.05 | |
| *Y537S* | 0.02 ± 0.02* | 0.27 | 0.84 ± 0.08 | |
| *D538G* | ND* | 0.013 | 0.87 ± 0.13 | |
| **Stabilizers (SERM-Like)** | | | | |
| **Ligand** | **IC_50_ (nM)** | **R^2^** | **Maxima (5 µM)** | |
| **4-Hydroxytamoxifen (4OHT)** | | | | |
| *WT* | 1.56 ± 0.05 | 0.92 | 2.23 ± 0.24 | |
| *Y537S* | 12.42± 0.075 | 0.9 | 2.25 ± 0.23 | |
| *D538G* | ND | 0.11 | 0.93 ± 0.37 | |
| **RU39411** | | | | |
| *WT* | ND | 0.28 | 2.11 ± 0.36 | |
| *537S* | 4.33 ± 0.10 | 0.7 | 2.01 ± 0.16 | |
| *538G* | ND | 0.287 | 1.18 ± 0.15 | |
| **Laso-Stabilizer (LA-Stab)** | | | | |
| *WT* | 1.239 ± 0.06 | 0.9143 | 2.42 ± 0.19 | |
| *537S* | 10.62 ± 0.07 | 0.934 | 2.61 ± 0.25 | |
| *538G* | ND | 0.07 | 1.17 ± 0.22 | |
| **Clomiphene (Clom)** | | | | |
| *WT* | 29.57 ± 0.11 | 0.81 | 1.92 ± 0.33 | |
| *537S* | 296.5 ± 0.12 | 0.788 | 1.66 ± 0.20 | |
| *538G* | 85.64 ± 0.08 | 0.35 | 0.79 ± 0.12 | |
| **Neutral** | | | | |
| **Ligand** | **IC_50_ (nM)** | **R^2^** | | **Maxima (5 µM)** |
| **Lasofoxifene (Laso)** | | | | |
| *WT* | ND | 0.48 | 0.9 ± 0.08 | |
| *Y537S* | 1.19 ± 0.05 | 0.55 | 1.48 ± 0.22 | |
| *D538G* | ND | 0.27 | 0.95 ± 0.27 | |
| **Degraders (SERD-Like)** | | | | |
| **Ligand** | **IC_50_ (nM)** | **R^2^** | **Maxima (5 µM)** | |
| **RU58668** | | | | |
| *WT* | 2.25 ± 0.04 | 0.92 | 0.2 ± 0.03 | |
| *537S* | 41.64 ± 0.06 | 0.90 | 0.25 ± 0.03 | |
| *538G* | 51.75 ± 7.00 | 0.8 | 0.37 ± 0.13 | |
| **Fulvestrant (ICI)** | | | | |
| *WT* | 0.40 ± 0.02 | 0.95 | 0.29 ± 0.04 | |
| *Y537S* | 3.76 ± 0.034 | 0.92 | 0.33 ± 0.08 | |
| *D538G* | ND | 0.19 | 0.67 ± 0.37 | |
| **GDC0927** | | | | |
| *WT* | 0.13 ± 0.03 | 0.72 | 0.33 ± 0.04 | |
| *537S* | 2.18 ± 0.03 | 0.90 | 0.36 ± 0.06 | |
| *538G* | 1.88 ± 0.04 | 0.75 | 0.47 ± 0.15 | |
| **OP1074** | | | | |
| *WT* | 1.09 ± 0.03 | 0.90 | 0.36 ± 0.05 | |
| *537S* | 14.07 ± 0.03 | 0.89 | 0.56 ± 0.04 | |
| *538G* | 23.35 ± 0.06 | 0.67 | 0.63 ± 0.08 | |
| **Bazedoxifene (BZA)** | | | | |
| *WT* | 0.87 ± 0.03 | 0.85 | 0.48 ± 0.06 | |
| *537S* | ND | 0.32 | 0.81 ± 0.13 | |
| *538G* | ND | 0.38 | 0.67 ± 0.11 | |
| **Pipendoxifene (PIP)** | | | | |
| *WT* | 0.77 ± 0.03 | 0.88 | 0.43 ± 0.08 | |
| *537S* | 6.28 ± 0.04 | 0.81 | 0.45 ± 0.06 | |
| *538G* | 18.29 ± 0.06 | 0.71 | 0.60 ± 0.07 | |
| **Laso-Degrader (LADeg)** | | | | |
| *WT* | 1.13 ± 0.03 | 0.78 | 0.56 ± 0.08 | |
| *Y537S* | ND | ND | 0.89 ± 0.10 | |
| *D538G* | ND | 0.09 | 1.06 ± 0.34 | |
| **OP1154** | | | | |
| *WT* | 48.92 ± 0.06 | 0.73 | 0.55 ± 0.09 | |
| *537S* | 19.85 ± 0.04 | 0.61 | 1.54 ± 0.14 | |
| *538G* | ND | 0.02 | 0.54 ± 0.09 | |
| **AZD9496** | | | | |
| *WT* | 8.78 ± 0.05 | 0.63 | 0.58 ± 0.08 | |
| *537S* | ND | 0.20 | 1.1 ± 0.18 | |
| *538G* | ND | 0.30 | 0.74 ± 0.05 | |
| **Raloxifene (RAL)** | | | | |
| *WT* | 0.85 ± 0.03 | 0.79 | 0.59 ± 0.07 | |
| *537S* | ND | 0.37 | 0.83 ± 0.06 | |
| *538G* | ND | ND | 0.85 ± 0.14 | |
| **LSZ102** | | | | |
| *WT* | 1.15 ± 0.03 | 0.73 | 0.60 ± 0.06 | |
| *537S* | ND | 0.40 | 1.30 ± 0.10 | |
| *538G* | ND | 0.58 | 0.73 ± 0.08 | |
| **GDC0810** | | | | |
| *WT* | 26.1 ± 0.05 | 0.69 | 0.60 ± 0.06 | |
| *537S* | ND | 0.59 | 1.38 ± 0.10 | |
| *538G* | ND | ND | 0.78 ± 0.10 | |
