# Supplementary material for: Stereospecific lasofoxifene derivatives reveal the interplay between estrogen receptor alpha stability and antagonistic activity in ESR1 mutant breast cancer cells
Source: eLife. 2022 May 16;11:e72512. doi: 10.7554/eLife.72512 (PMC9177151; doi:10.7554/eLife.72512)
Supplement: Figure 4—source data 1. — The SEM values for IC50s were all within 20% except for Ral with WT (21.4%) and OHT for D538G (26.2%). [file elife-72512-fig4-data1.docx]

| **Ligand** | **Protein** | **IC_50_ (nM)** | **R^2^** |
| --- | --- | --- | --- |
| **ICI** | WT | 17.8 | 0.90 |
|  | Y537S | 42.9 | 0.92 |
|  | D538G | 38.1 | 0.93 |
| **GDC0927** | WT | 254 | 0.86 |
|  | Y537S | 1,961 | 0.74 |
|  | D538G | 1,273 | 0.82 |
| **LA-Deg** | WT | 49.9 | 0.93 |
|  | Y537S | 334 | 0.93 |
|  | D538G | 240 | 0.93 |
| **LA-Stab** | WT | 70.4 | 0.92 |
|  | Y537S | 344 | 0.91 |
|  | D538G | 266 | 0.90 |
| **RAL** | WT | 32.4 | 0.92 |
|  | Y537S | 189 | 0.90 |
|  | D538G | 166 | 0.93 |
| **AZD9496** | WT | 1.7 | 0.92 |
|  | Y537S | 12.2 | 0.95 |
|  | D538G | 13.5 | 0.95 |
| **4OHT** | WT | 51.0 | 0.90 |
|  | Y537S | 255 | 0.994 |
|  | D538G | 202 | 0.95 |
