# Supplementary material for: Stereospecific lasofoxifene derivatives reveal the interplay between estrogen receptor alpha stability and antagonistic activity in ESR1 mutant breast cancer cells
Source: eLife. 2022 May 16;11:e72512. doi: 10.7554/eLife.72512 (PMC9177151; doi:10.7554/eLife.72512)
Supplement: Figure 4—source data 2. — The SEM values for IC50s were all within 50% except for D538G with AZD9496 (52%). [file elife-72512-fig4-data2.docx]

| **Ligand** | **Protein** | **EC_50_ (nM)** | **R^2^** |
| --- | --- | --- | --- |
| **ICI + 1 nM E2** | WT | 197.8 | 0.95 |
|  | Y537S | 231.7 | 0.95 |
|  | D538G | 433.9 | 0.68 |
