# Supplementary material for: Stereospecific lasofoxifene derivatives reveal the interplay between estrogen receptor alpha stability and antagonistic activity in ESR1 mutant breast cancer cells
Source: eLife. 2022 May 16;11:e72512. doi: 10.7554/eLife.72512 (PMC9177151; doi:10.7554/eLife.72512)
Supplement: Figure 5—source data 1. — Data were normalized by cell count. [file elife-72512-fig5-data1.docx]

**Figure 5-source data 1:** Ligand and Mutational Influences on Estrogen Receptor Alpha Reporter Gene Transcription after 24 Hours. Data were normalized by cell count.

| **Hormone** | | | | |
| --- | --- | --- | --- | --- |
| **Ligand** | **IC_50_ (nM)** | **Fold Change vs WT** | **R^2^** | **Maxima (5 µM)** |
| **Estradiol (E2)** | | | | |
| *WT* | 0.004 ± 0.08 |  | 0.84 | 1.47 ± 0.16 |
| *Y537S* | 158.1 ± 0.20 | 39,500 | 0.71 | 1.14 ± 0.18 |
| *D538G* | ND |  | ND | 1.15 ± 0.14 |
| **Stabilizers (SERM-Like)** | | | | |
| **Ligand** | **IC_50_ (nM)** |  | **R^2^** | **Maxima (5 µM)** |
| **4-Hydroxytamoxifen (4OHT)** | | | | |
| *WT* | 0.79 ± 0.07 |  | 0.99 | 0.29 ± 0.05 |
| *Y537S* | 5.10 ± 0.18 | 6.46 | 0.81 | 0.73 ± 0.11 |
| *D538G* | 6.85 ± 0.60 | 8.67 | 0.88 | 0.42 ± 0.05 |
| **RU39411** | | | | |
| *WT* | 0.22 ± 0.07 |  | 0.99 | 0.14 ± 0.02 |
| *537S* | 3.96 ± 0.55 | 5.01 | 0.90 | 0.32 ± 0.02 |
| *538G* | 0.26 ± 0.53 | 1.18 | 0.70 | 0.46 ± 0.13 |
| **Laso-Stabilizer (LA-Stab)** | | | | |
| *WT* | 0.28± 0.06 |  | 0.98 | 0.33 ± 0.03 |
| *537S* | 22.97 ± 0.44 | 81.92 | 0.97 | 0.51 ± 0.08 |
| *538G* | 12.84 ± 0.58 | 45.86 | 0.74 | 0.43 ± 0.05 |
| **Neutral** | | | | |
| **Ligand** | **IC_50_ (nM)** |  | **R^2^** | **Maxima (5 µM)** |
| **Lasofoxifene (Laso)** | | | | |
| *WT* | 0.20 ± 0.07 |  | 0.99 | 0.33 ± 0.09 |
| *Y537S* | 2.88 ± 0.34 | 14.4 | 0.95 | 0.57 ± 0.07 |
| *D538G* | 0.55 ± 0.70 | 2.75 | 0.90 | 0.34 ± 0.04 |
| **Degraders (SERD-Like)** | | | | |
| **Ligand** | **IC_50_ (nM)** |  | **R^2^** | **Maxima (5 µM)** |
| **RU58668** | | | | |
| *WT* | 0.92 ± 0.08 |  | 0.96 | 0.21 ± 0.01 |
| *537S* | 11.30 ± 0.61 | 12.28 | 0.99 | 0.37 ± 0.08 |
| *538G* | 4.34 ± 0.57 | 4.72 | 0.70 | 0.34 ± 0.08 |
| **Fulvestrant (ICI)** | | | | |
| *WT* | 0.35 ± 0.06 |  | 0.99 | 0.27 ± 0.05 |
| *Y537S* | 6.50 ± 0.39 | 18.57 | 0.94 | 0.56 ± 0.07 |
| *D538G* | 0.57 ± 0.07 | 1.63 | 0.92 | 0.32 ± 0.03 |
| **GDC0927** | | | | |
| *WT* | 0.03 ± 0.08 |  | 0.96 | 0.27 ± 0.01 |
| *537S* | 0.95 ± 0.51 | 31.67 | 0.90 | 0.42 ± 0.07 |
| *538G* | ND |  | ND | 0.71 ± 0.17 |
| **OP1074** | | | | |
| *WT* | 0.17 ± 0.05 |  | 0.93 | 0.30 ± 0.09 |
| *537S* | 4.91 ± 0.31 | 28.88 | 0.87 | 0.59 ± 0.06 |
| *538G* | 0.52 ± 0.63 | 3.06 | 0.71 | 0.37 ± 0.05 |
| **Bazedoxifene (BZA)** | | | | |
| *WT* | 0.80 ± 0.10 |  | 0.99 | 0.17 ± 0.01 |
| *537S* | 11.61 ± 0.67 | 14.51 | 0.97 | 0.30 ± 0.05 |
| *538G* | 6.15 ± 0.66 | 7.69 | 0.80 | 0.34 ± 0.10 |
| **Pipendoxifene (PIP)** | | | | |
| *WT* | 0.93 ± 0.08 |  | 0.96 | 0.19 ± 0.02 |
| *537S* | 10.60 ± 0.64 | 11.40 | 0.97 | 0.33 ± 0.04 |
| *538G* | 7.45 ± 0.69 | 8.01 | 0.78 | 0.29 ± 0.07 |
| **Laso-Degrader (LA-Deg)** | | | | |
| *WT* | 0.23 ± 0.07 |  | 0.99 | 0.34 ± 0.04 |
| *Y537S* | 3.30 ± 0.38 | 14.35 | 0.97 | 0.56 ± 0.07 |
| *D538G* | 2.76 ± 0.62 | 12.00 | 0.76 | 0.4 ± 0.06 |
| **OP1154** | | | | |
| *WT* | 1.15 ± 0.65 |  | 0.99 | 0.28 ± 0.04 |
| *537S* | 31.15 ± 0.04 | 27.09 | 0.99 | 0.55 ± 0.04 |
| *538G* | 8.74 ± 0.85 | 7.60 | 0.92 | 0.25 ± 0.03 |
| **AZD9496** | | | | |
| *WT* | 1.53 + 0.09 |  | 0.94 | 0.22 + 0.01 |
| *537S* | 14.42 ± 0.56 | 9.42 | 0.95 | 0.38 ± 0.07 |
| *538G* | 2.67 ± 0.64 | 1.75 | 0.80 | 0.38 ± 0.11 |
| **Raloxifene (RAL)** | | | | |
| *WT* | 0.11 ± 0.08 |  | 0.99 | 0.16 ± 0.02 |
| *537S* | 2.16 ± 0.67 | 19.63 | 0.99 | 0.33 ± 0.08 |
| *538G* | 2.29 + 0.67 | 20.81 | 0.891 | 0.32 + 0.03 |
| **LSZ102** | | | | |
| *WT* | 0.34 ± 0.08 |  | 0.97 | 0.28 ± 0.10 |
| *537S* | 7.03 ± 0.30 | 20.67 | 0.94 | 0.64 ± 0.15 |
| *538G* | ND |  | ND | 0.50 ± 0.19 |
| **GDC0810** | | | | |
| *WT* | 4.49± 0.08 |  | 0.99 | 0.34 ± 0.06 |
| *537S* | 56.39 ± 0.32 | 12.54 | 0.96 | 0.64 ± 0.06 |
| *538G* | 3.74 ± 0.52 | 0.83 | 0.60 | 0.41 ± 0.10 |
