# Supplementary material for: Stereospecific lasofoxifene derivatives reveal the interplay between estrogen receptor alpha stability and antagonistic activity in ESR1 mutant breast cancer cells
Source: eLife. 2022 May 16;11:e72512. doi: 10.7554/eLife.72512 (PMC9177151; doi:10.7554/eLife.72512)
Supplement: Figure 6—source data 1. [file elife-72512-fig6-data1.docx]

**Figure 6-supplemental table 1:** SERM-agonist activities in uterine epithelial cells measured by induction of alkaline phosphatase.

| **Ishikawa** | | | |
| --- | --- | --- | --- |
| **Treatment** | **EC_50_** | **R^2^** | **Max (1μM)** |
| Veh (DMSO) |  |  | 0.33 + 0.01 |
| 1 nM E2 |  |  | 2.35 + 0.05 |
| ICI | N.D. | N.D. | 0.32 + 0.01 |
| LA-Deg | N.D. | N.D. | 0.47 + 0.03 |
| Laso | 0.05 + 0.02 | 0.80 | 0.96 + 0.40 |
| LA-Stab | 0.68 + 0.09 | 0.91 | 1.49 + 0.03 |
| Z-Endoxifen | 5.14 + 1.23 | 0.92 | 1.04 + 0.02 |
| 4OHT | 0.32 + 0.04 | 0.7 | 2.14 + 0.22 |
