# Supplementary material for: Stereospecific lasofoxifene derivatives reveal the interplay between estrogen receptor alpha stability and antagonistic activity in ESR1 mutant breast cancer cells
Source: eLife. 2022 May 16;11:e72512. doi: 10.7554/eLife.72512 (PMC9177151; doi:10.7554/eLife.72512)
Supplement: Figure 8—source data 1. [file elife-72512-fig8-data1.docx]

**Figure 8-source data 1:** X-ray Crystal Structure Data Collection and Refinement

|  | **ERα LBD Y537S BZA** | **ERα LBD RAL** | **ERα LBD Y537S RAL** | **ERα LBD Y537S 4OHT** |
| --- | --- | --- | --- | --- |
| PDB ID | 6PSJ | 7KBS | 7UJC | 7UJ8 |
| **Data Collection** | | | | |
| Space Group | C2 | C2 | C2 | C2 |
| a, b, c (Å) | 103.19, 56.49, 87.63 | 101.802, 57.879, 90.00 | 102.56, 57.84, 87.94 | 104.20, 55.07, 97.48 |
| α, β, γ (°) | 90.00, 104.32, 90.00 | 90.00, 120.93, 90.00 | 90.00, 103.74, 90.00 | 90.00, 113.48, 90.00 |
| Resolution Range (Å) | 29.14 - 1.80 | 30.86 – 1.83 | 29.18 – 1.79 | 39.00 – 2.39 |
| Number of Reflections (all/unique) | 155,419/43,172 | 140,900/39,139 | 141,501/39,306 | 64,695/17,971 |
| Completeness (Highest Resolution) | 95.0 (82.8) | 98.4/89.2 | 99.4/82.2 | 96.5/96.9 |
| Redundancy | 3.6 | 3.5 | 3.6 | 3.1 |
| CC^1/2^ (Highest Resolution) | 0.61 | 0.55 | 0.51 | 0.751 |
| **Refinement** | | | | |
| R_work_/R_free_ | 18.5/21.5 | 17.13/20.97 | 19.15/23.00 | 24.2/27.1 |
| No. Atoms | 3,486 | 4,193 | 3,892 | 3,429 |
| Water Molecules | 360 | 338 | 308 | 58 |
| Ligand Molecules | 70 | 68 | 68 | 48 |
| Bond Lengths (Å) | 0.03 | 0.007 | 0.015 | 0.006 |
| Bond Angles (°) | 1.493 | 1.005 | 1.104 | 0.694 |
| **Ramachandaran Plot Statistics** | | | | |
| Preferred Number (%) | 99.32 | 98.69 | 99.30 | 97.81 |
| Additional Allowed (%) | 0.68 | 1.31 | 0.70 | 2.19 |
| Outliers (%) | 0 | 0 | 0 | 0 |

|  | **ERα LBD LA-Stab** | **ERα LBD Y537S LA-Stab** | **ERα LBD LA-Deg** | **ERα LBD Y537S LA-Deg** |
| --- | --- | --- | --- | --- |
| PDB ID | 7UJM | 7UJY | 7UJF | 7UJW |
| **Data Collection** | | | | |
| Space Group | P3_2_ | P3_2_ | P3_2_ | P1 |
| a, b, c (Å) | 58.467, 58.467, 275.346 | 58.34, 58.34, 274.65 | 58.66, 58.66, 276.37 | 53.949, 58.974, 88.993 |
| α, β, γ (°) | 90.00, 90.00, 120.00 | 90.00, 90.00, 120.00 | 90.00, 90.00, 120.00 | 91.46, 102.06, 117.23 |
| Resolution Range (Å) | 44.33 – 1.80 | 49.69 – 1.70 | 47.68 – 1.70 | 47.59 – 2.60 |
| Number of Reflections (all/unique) | 107,823/27,646 | 105,602/25,562 | 117,080/23,893 | 50,416/28,009 |
| Completeness (Highest Resolution) | 98.9/80.0 | 99.9/91.88 | 100/81.62 | 96.30/87.13 |
| Redundancy | 3.9 | 4.5 | 4.9 | 1.8 |
| CC^1/2^ (Highest Resolution) | 0.726 | 0.603 | 0.669 | 0.601 |
| **Refinement** | | | | |
| R_work_/R_free_ | 18.97/23.29 | 21.0/24.4 | 19.5/23.9 | 21.2/26.3 |
| No. Atoms | 8,414 | 8,168 | 8,518 | 6,716 |
| Water Molecules | 684 | 630 | 823 | 86 |
| Ligand Molecules | 128 | 128 | 128 | 128 |
| Bond Lengths (Å) | 0.011 | 0.027 | 0.009 | 0.004 |
| Bond Angles (°) | 1.261 | 1.522 | 1.218 | 0.972 |
| **Ramachandaran Plot Statistics** | | | | |
| Preferred Number (%) | 98.93 | 98.90 | 98.42 | 96.33 |
| Additional Allowed (%) | 1.07 | 1.10 | 1.58 | 3.67 |
| Outliers (%) | 0 | 0 | 0 | 0 |
